# Proton and non-proton activation of ASIC channels

**DOI:** 10.1371/journal.pone.0175293

**Published:** 2017-04-06

**Authors:** Ivan Gautschi, Miguel Xavier van Bemmelen, Laurent Schild

**Affiliations:** Department of Pharmacology and Toxicology, Faculty of Biology and Medicine, University of Lausanne, Lausanne, Switzerland; Indiana University School of Medicine, UNITED STATES

## Abstract

The Acid-Sensing Ion Channels (ASIC) exhibit a fast desensitizing current when activated by pH values below 7.0. By contrast, non-proton ligands are able to trigger sustained ASIC currents at physiological pHs. To analyze the functional basis of the ASIC desensitizing and sustained currents, we have used ASIC1a and ASIC2a mutants with a cysteine in the pore vestibule for covalent binding of different sulfhydryl reagents. We found that ASIC1a and ASIC2a exhibit two distinct currents, a proton-induced desensitizing current and a sustained current triggered by sulfhydryl reagents. These currents differ in their pH dependency, their sensitivity to the sulfhydryl reagents, their ionic selectivity and their relative magnitude. We propose a model for ASIC1 and ASIC2 activity where the channels can function in two distinct modes, a desensitizing mode and a sustained mode depending on the activating ligands. The pore vestibule of the channel represents a functional site for binding non-proton ligands to activate ASIC1 and ASIC2 at neutral pH and to prevent channel desensitization.

## Introduction

Acid-sensing ion channels (ASICs) are ligand-gated channels activated by protons. ASICs belong to the ENaC/degenerins family of ion channels that includes constitutively active channels, peptide-gated channels, and mechanosensitive channels. Six subtypes of ASIC are expressed in the vertebrate central and peripheral nervous systems. ASIC1 and ASIC3 are expressed in primary afferent sensory neurons where they initiate nociceptive signals in response to a drop in extracellular pH associated with ischemic or inflammatory conditions [1, 2]. Beside its role in pain sensation, ASIC1 was reported to promote learning and fear conditioning [2]. ASIC-dependent excitatory postsynaptic currents (EPSCs) have been recorded *in vivo* although they remain quite small compared to glutamate-dependent EPSCs for instance [2].

ASIC channels are cation-selective and voltage-insensitive channels. The extracellular pH required for half-maximal activation of ASIC channels range from 4.5 to 6.8 depending on the subtype. The current elicited by protons is transient and followed by a desensitization that maintains the channel in a non-conducting state until the pH returns to physiological values. There is no specific high affinity pharmacological blocker of ASIC channels except toxins from spider (PcTx), sea anemone (APETx2), or snakes (mambalgin) [3] [4] [5]. MitTx, a toxin isolated from Texas coral snake venom produces excruciating pain in humans and activates ASIC1 in nociceptor cells [6].

Still fundamental questions remain unresolved about ASIC function *in vivo* that include the unphysiological pH values used to activate the channels or the transient nature of the proton-evoked ASIC current. Assuming that protons are the sole physiological activators, ASIC activity *in vivo* would require large and rapid pH fluctuations in the extracellular milieu. Presynaptic stimulation *in vivo* results in both neurotransmitter release and acidification of the synaptic space, but it is still not established whether such a pH drop is sufficient to activate ASICs [7, 8].

Several recent experiments indicate that ASIC channels can be activated by ligands other than protons. Exposing ASIC1 to nanomolar concentrations of MitTx activates a large sustained and non-desensitizing current at neutral pH that exceeds in magnitude the maximal current evoked [6]. The polyamines GMQ (2-guandine-4-methylquinazoline) or agmatine similarly activate ASIC3 at neutral pH [9]. Neuropeptides potentiate a H^+^-gated current carried by ASIC and induce a sustained current [10]. Furthermore, covalent modification with sulfhydryl reagents of cysteine residues introduced in the pore vestibule of ASIC1 triggers a sustained activity [11–13]

In contrast to ASIC channels, the other members of the ENaC/Deg ion channel family elicit sustained currents at physiological pHs. The mollusk FMRF-gated channels are activated by peptides and produce sustained currents [14]. ENaC is a constitutively active channel when present at the cell surface. In this study, we have used mutated ASIC channels with cysteine residues introduced in the extracellular vestibule to study the ASIC sustained currents after covalent binding with different MTS reagents [11]. A careful analysis of the pH dependence of these MTS-induced currents allowed us to propose a model for a sustained ASIC activity at a physiological pH.

## Results

We have generated ASIC1a-G430C, an ASIC1a mutant with a G430C cysteine substitution in the first N-terminal turn of the second transmembrane α helix that lines the extracellular vestibule of the channel pore. G430 of hASIC1a is located one helical turn upstream of a channel-activating site referred in the literature as the degenerin site [1]. We have used different MTS-reagents (see S1 Fig) for covalent modification of the G430C, and analyzed their effects on G430C-mediated currents. We verified that neither MTSET nor the G430C substitution had any effect on the pH-dependence of wt ASIC1a activation (S2 Fig).

### Accessibility of MTS-reagents to G430C

**Fig 1:**
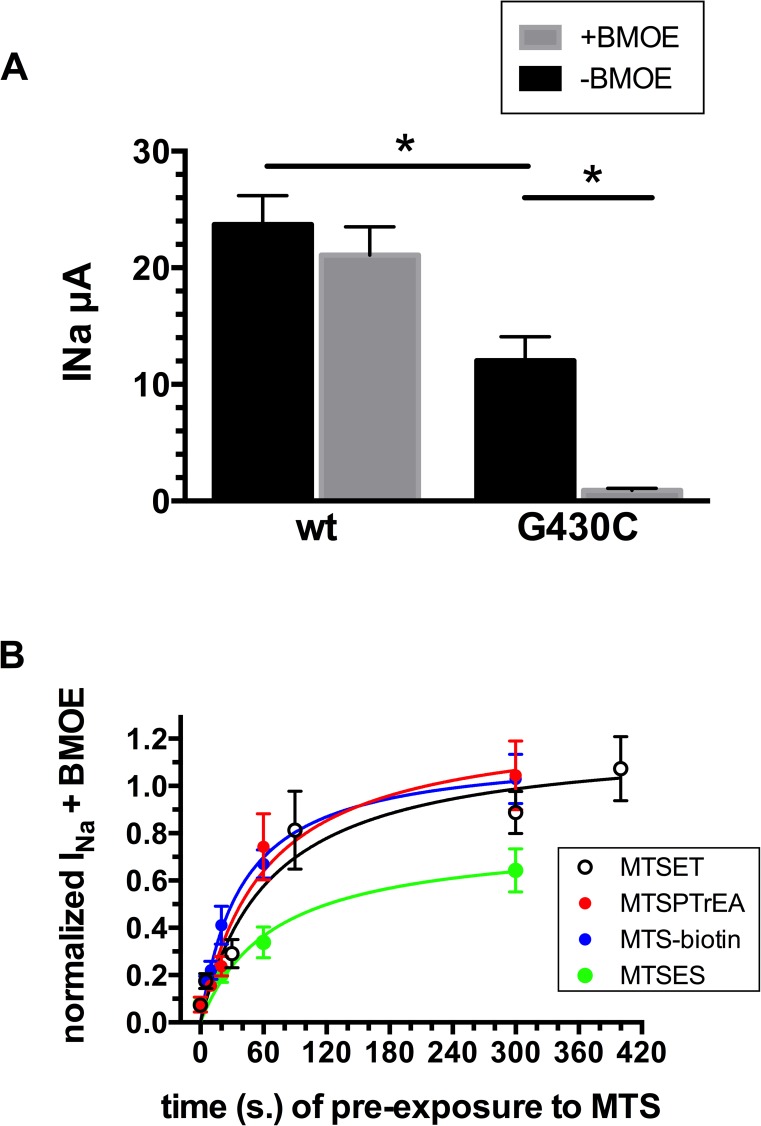
Accessibility of MTSET, MTSPTrEA, MTSEA-biotin and MTSES to the cysteine at position G430 of ASIC1a. **A**. Current measurements elicited at pH 5.5 in oocytes expressing ASIC1a wt (n = 72) or ASIC1a-G430C (n = 70) before (-BMOE, n = 77) or after (+BMOE, n = 65) 5 min. of incubation with 2 mM BMOE. * denotes p< 0.01. **B.** Relation between the ASIC1a-G430C peak current measured in the presence of 2 mM BMOE, and the time of pre-exposure to 10 μM of either MTSET, MTSPTrEA, MTSEA-biotin or MTSES. Current values were normalized for the peak current measured after 10 min. exposure of MTSET, MTSPTrEA, MTSEA-biotin or MTSES (10 μM), in the absence of BMOE. Each point represents the mean (m±SE) of 7 to 11 measurements.

We have first verified the ability of MTS reagents to coordinate covalently G430C. We took advantage of the ability of the maleimide crosslinker BMOE (2 mM) to irreversibly block ASIC1a-G430C but not ASIC1a wt [15]. Fig 1A shows the specific block of G430C compared to wt; the G430C mutant exhibits a slightly lower current expression (see also Table 1). The different MTS reagents tested did not inhibit ASIC1-G430C current (Table 1). We first tested the accessibility of MTSET, MTSPTrEA, MTSEA-biotin and MTSES to G430C, and their ability to covalently modify G430C. We performed competition experiments based on the hypothesis that MTS-reagents bound to G430C should prevent the block of ASIC1a-G430C by BMOE (see methods). Fig 1B shows the magnitude of ASIC1a current in the presence of BMOE as a function of the duration of channel pre-exposure to MTS-reagents. A pre-exposure to 10 μM MTSET, MTSPTrEA, or MTSEA-biotin for 5–6 minutes is sufficient to elicit the maximal ASIC1a current in the presence of BMOE, and to prevent more than 90% of the ASIC1a-G430C block by BMOE. For MTSES the kinetics of current preservation from channel block by BMOE was similar to that of the other MTS-reagents, but the efficiency of MTSES in preventing this block seems to be slightly lower. These experiments show that the cysteine at position G430 in the external vestibule of ASIC1a is accessible to MTS-reagents for covalent modification.

**Table 1:** pH dependency of current activation and steady state desensitization (SSD) of ASIC1 G430C unbound or bound to MTS-reagents.

	I _max_ (μA)	Fractional I_sust_	pH_05_ I_sust_	pH_0.5_ I_desens._	pH_0.5_ SSD
ASIC1a wt (n = 41)	55.33±3.88	nd	nd	6.43 (6.41-6.46)	7.15 (7.15-7.16)
ASIC1a-G430C (n = 80)	15.37±1.33	nd	nd	6.34 (6.30-6.37)	7.08 (7.07-7.11)
G430C+MTSET (n = 19)	29.72±4.02*	0.10 ± 0.01	>7.0	6.70 (6.68-6.73)	7.17 (7.15-7.19)
G430C+MTSPTrEA (n = 29)	36.76±4.19*	0.44 ± 0.01	7.35 (7.27-7.42)	6.91 (6.88-6.94)	7.20 (7.18-7.21)
G430C+MTSBT (n = 31)	37.69±4.35*	0.23± 0.03	7.11 (6.94-7.28)	6.82 (6.73-6.91)	7.17 (7.14-7.20)
G430C+MTSPT (n = 13)	19.53±2.70	0.23± 0.02	7.29 (7.22-7.37)	6.78 (6.74-6.82)	7.17 (7.14-7.20)
G430C+MTSEA (n = 11)	20.37±3.12	0.05± 0.004	>7.0	6.85 (6.83-6.87)	7.08 (7.06-7.09)
G430C+MTSEA-biotin (n = 26)	43.13±4.61*	0.33± 0.02	>7.0	7.01 (6.99-7.03)	7.19 (7.16-7.22)
G430C+MTSES (n = 42)	25.25±2.74*	nd	nd	6.80 (6.77-6.83)	7.11 (7.10-7.12)

Oocytes expressing ASIC1a wt or G430C were analyzed for expression of ASIC1a currents elicited at pH 5.5. Oocytes expressing G430C were pre-incubated with MTS-reagents at pH 7.8 for 10 minutes before current measurements. I_max_ represents the maximal inward peak current, I_sust_ and I_desens_ denote respectively the sustained current and the desensitizing current. Fractional I_sust_ denotes the fraction of the total ASIC1a current that corresponds to I_sust_. I_sust_ for ASIC1a wt, G430C unbound or bound to MTSES (nd) was not detected, and the pH_0.5_ of I_sust_ could not be fitted but was >pH 0.7. The pH_0.5_ for current activation was determined by a one-component non-linear fit of the current values.

* denotes statistical significant difference with the mean value obtained for ASIC1a-G430C (one-way ANOVA), pH values in parenthesis represent 95% confidence intervals obtained for the fit.

### Effects of MTS-reagents on ASIC1a currents

**Fig 2:**
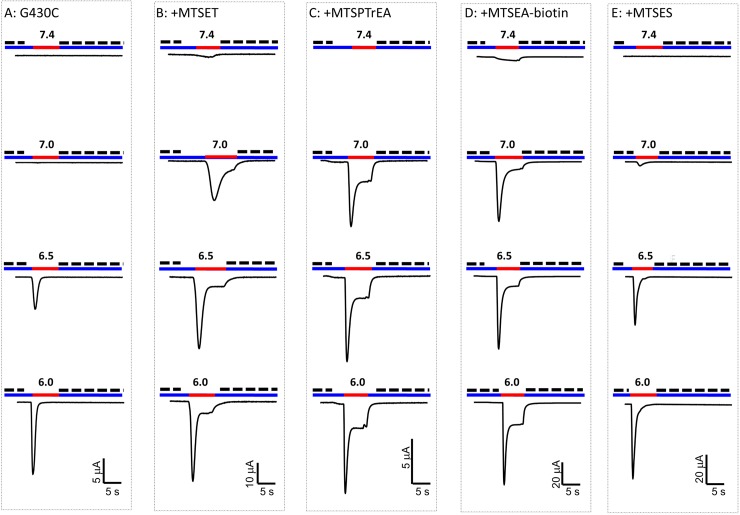
Representative tracings of ASIC1a-G430C currents elicited at different pH values after covalent modification with MTS-reagents. Oocytes expressing ASIC1-G430C were perfused at pH7.8 (blue line) and ASIC1a currents were elicited by a pH change (red line) to 7.4, 7.0, 6.5, or 6.0; amiloride (300 μM, dashed line) was added to bath upon pH return to 7.8. In (**A**) the oocyte expressing ASIC1a-G430C was not pre-incubated with MTS, while in all the other conditions oocytes were pre-incubated at pH 7.8 for 10 min with 100 μM of one of the following compounds: MTSET (**B**), MTSPTrEA (**C**), MTSEA-biotin (**D**), or MTSES (**E**).

Fig 2 shows representative tracings of the ASIC1a-G430C-mediated inward currents elicited at different pH values, before and after covalent modification of the channel by exposure during 10 min to 100 μM of MTSET, MTSPTrEA, MTSEA-biotin, or MTSES. For the unmodified G430C, a fast transient current was observed at pHs below 7.0. After modification, an amiloride-sensitive, non-desensitizing inward current was already detected at pH 7.4 for MTSET, MTSPTrEA and MTSEA-biotin, but not for MTSES. At pH 7.0 and below, robust transient inward currents were observed followed by a non-desensitizing sustained current (I_sust_). After modification of G430C by negatively charged MTSES, no sustained current could be detected. These tracings suggest that the inward current elicited by protons on the G430C channel covalently modified by MTS-reagents results from two distinct currents, a non-desensitizing sustained current (I_sust._) and a fast desensitizing current (I_desens_).

**Fig 3:**
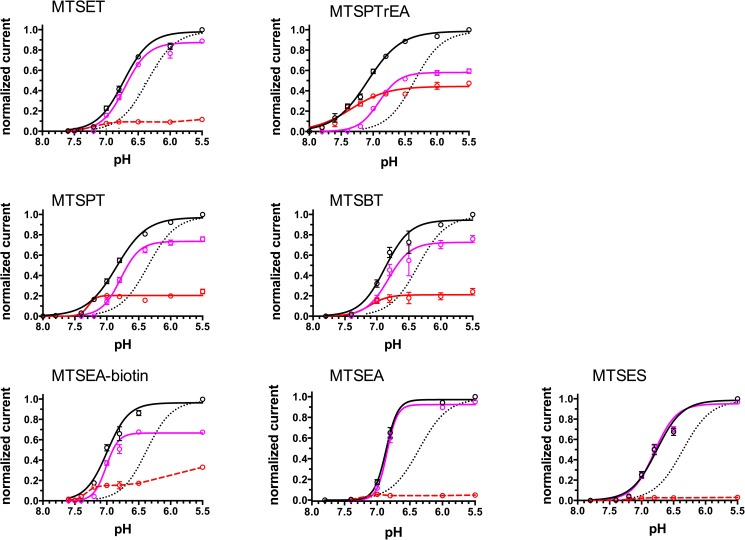
pH-dependence of activation of ASIC1a-G430C currents after channel modification by MTS-reagents. Currents recorded in oocytes expressing ASIC1a-G430C and incubated beforehand with MTSET, MTSPTrEA, MTSPT, MTSBT, MTSEA-biotin, MTSEA, MTSES (100 μM) during 10 min. at pH 7.8. Currents are elicited by acidic pH changes ranging from 7.8 to 5.5. Black circles and black solid lines represent the total inward current, pink circles and lines represent the desensitizing current (I_desens_), red circles and lines the sustained current (I_sust_). The lines represent the best non-linear fit for the pH-dependence of current activation. For comparison, the dashed line represents the pH dependence of activation of ASIC1-G430C without pre-incubation with MTS-reagents obtained from data shown in S2 Fig. Each current value was normalized for the maximal total inward current elicited at pH 5.5. Each symbol represents the mean ± SE of 10 to 53 measurements.

In Fig 3 we show the analysis of the total current elicited after modification of ASIC1a-G430C by the different MTS-reagents at different pHs. The maximal ASIC1a current (I_max_) elicited by protons is represented as the sum of a transient desensitizing current (I_desens_) and a sustained current (I_sust_). After modification by MTSET, an alkaline shift of the pH-dependency (pH_0.5_) of ASIC1a-G430C activation was observed for both I_desens_ and I_sust_ currents; the I_sust_ levels off at around pH 7.0, plateauing at 10% of I_max_, the major fraction of this current accounting for I_desens_. A similar analysis was performed for the ASIC1a-G430C currents after modification by MTSPTrEA: compared to MTSET, the alkaline shift of pH_0.5_ of I_sust_ activation was more pronounced, whereas the pH_0.5_ values of I_desens_ were comparable for the two MTS-reagents; after modification with MTSPTrEA, I_sust_ represented up to 45% of the I_max_ at pHs for maximal activation (see also Table 1). The pH_0.5_ for activation of the ASIC1a-G430C currents was measured after modification by MTSPT and MTSBT and show values for I_sust_ between those obtained for MTSET and MTSPTrEA, as for the relative magnitude of I_sust_ (Table 1). The effect of MTSEA-biotin on both the pH_0.5_ and the magnitude of I_sust_ was comparable to that of MTSBT and MTSPT. Modification of ASIC1a-G430C with MTSEA had only minimal effects on I_sust_ and modification by MTSES did not induce any detectable I_sust_. MTSES, like the other MTS-reagents, shifted the pH_0.5_ of the I_desens_ to a similar extent. The values for pH_0.5_ obtained for both I_desens_ and I_sust_ are summarized in Table 1: the pH_0.5_ for I_desens,_ of the non-modified ASIC1a-G430C was 6.55 ±0.02, similar to ASIC1a wt, but after modification by the different MTS reagents including MTSES, the pH_0.5_ for I_desens_ was on average 6.89 ± 0.08 (mean ± SD). The average pH_0.5_ for I_sust_, when detectable, varied among the different MTS-reagents and was on average around 7.29 ± 0.11 (mean ± SD). The fraction of I_max_ carried by the I_sust_ also varied greatly among the MTS used for G430C modification and ranged from zero for the MTSES to around 0.45 for the MTSPTrEA.

**Fig 4:**
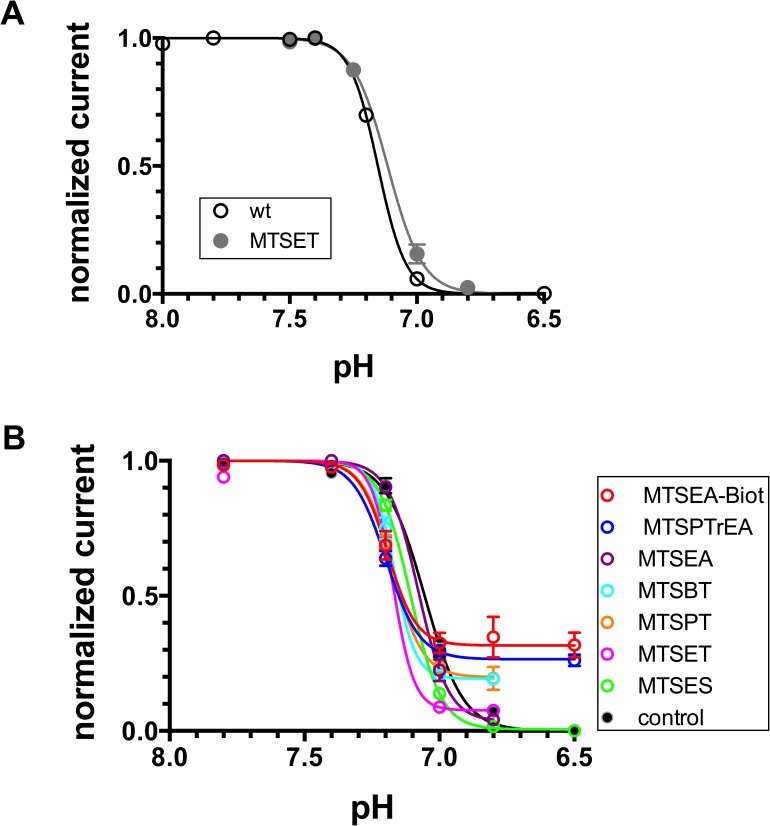
pH-dependence of steady-state desensitization (SSD). ASIC1a currents elicited by acidic pH 5.5, were measured after a 40 s. incubation at a conditioning pH ranging from 7.8 to 6.5. All currents were normalized for the maximal inward current elicited by pH drop from 7.8 to 5.5 (see methods). **A**. Current values obtained for SSD of ASIC1a-wt before (n = 22, open circles) and after incubation with MTSET (n = 10, closed circles). **B**. Current values obtained for SSD of ASIC1a-G430C before after pre-incubation with MTS reagents. Each symbol represents the mean ± SE for ASIC1a_G430C, either non-treated (control, n = 26), or pre-incubated for 10 min with 100 μM of either MTSET (n = 16), MTSPTrEA (n = 12), MTSBT (n = 4), MTSPT (n = 4), MTSEA (n = 6), MTSEA-biotin (n = 15), or MTSES (n = 8).

We have subsequently measured the pH_0.5_ of the steady-state desensitization (SSD) for the ASIC1-G430C channel, either unmodified or modified by the MTS-reagents. The pH_0.5_ obtained for the SSD of ASIC1a wt before and after exposure to MTSET are similar (Fig 4A). As expected, the desensitization was incomplete for the ASIC1-G430C bound to those MTS-reagents capable of inducing a sustained current (Fig 4B). The MTS reagents only produced a slight change in the pH_0.5_ of SSD, with a slight alkaline shift that did not exceed 0.1 pH unit (Table 1). The average pH_0.5_ for SSD of G430C was 7.08 ± 0.01 and 7.16 ± 0.02 after modification by MTS-reagents (Table 1).

**Fig 5:**
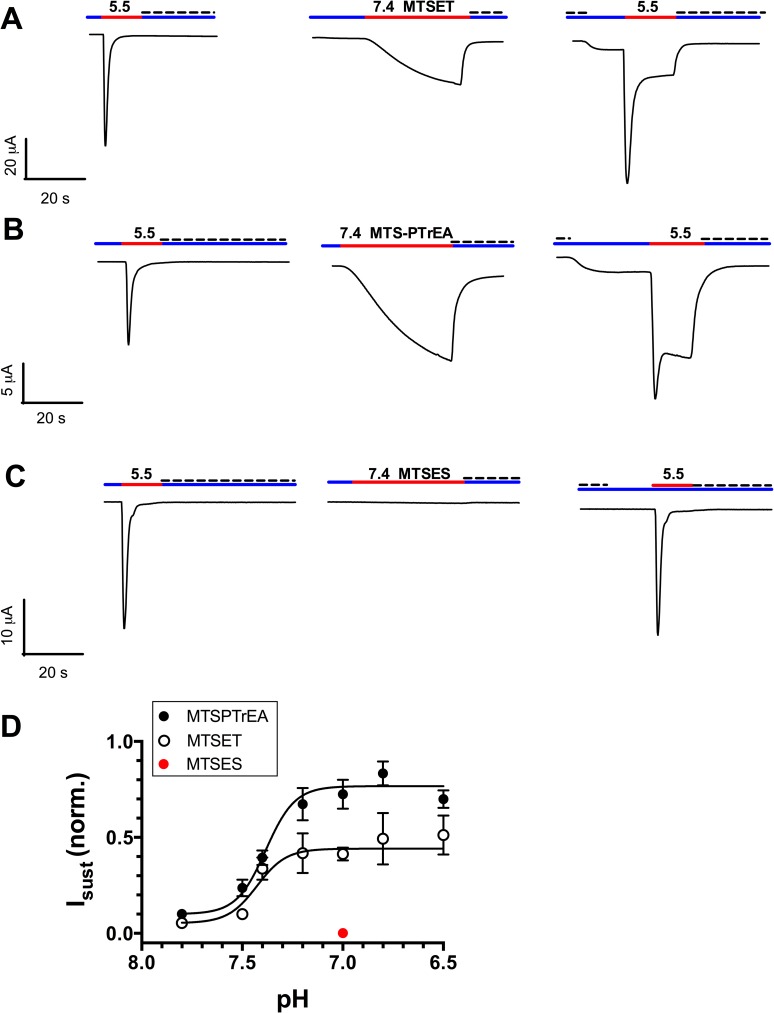
ASIC1a-G430C activation by MTS-reagents at neutral pH. **A-C**. Oocytes expressing G430C were perfused at pH 7.8 (blue line); current was first elicited at pH 5.5 (red line) before returning the extracellular pH 7.8 with amiloride (dashed line). Then, the extracellular pH was set at 7.4 or 7.0, and the oocytes perfused for 40 s. with solutions containing 100 μM (crosshatching red line) of either MTSET (**A**), MTSPTrEA (**B**), or MTSES (**C**); removal of MTS and addition of amiloride were done at pH7.8. A second acidic pulse at pH 5.5 (red line) was performed that included, sequentially, the removal of amiloride, the acidification at pH 5.5 and the re-addition of amiloride (300 μM) at pH 7.8. **D**. pH-dependence of ASIC1-G430C activation by MTSET (open circles) and MTSPTrEA (filled circles). Values obtained 30s. after addition of the MTS were normalized for the maximal current obtained during the second acidic pulse at pH 5.5. Values on the graph are mean ±SE from 15 to 53 measurements. Dashed lines represent the best fits for the pH dependency.

So far, we have considered the currents of ASIC1a-430C bound to MTS-reagents, which are elicited by H^+^. We also analyzed the pH_0.5_ of the activation of ASIC1a-G430C currents by the MTS-reagents. Fig 5 displays representative recordings of typical experiments in which a first acidic pH pulse was performed to ascertain the presence of active ASIC1a-G430C at the cell surface. The extracellular pH was then fixed at a pH value that sets the channel in the closed-resting or desensitizing states. Upon addition of MTSET (A) or MTSPTrEA (B) we recorded a robust, slowly activating and non-desensitizing inward current that was blocked by amiloride. A small but reproducible sustained current could still be measured at pH 7.8 after washout of amiloride. A second acidic pulse at pH 5.5 on the MTS-treated G430C resulted in a fast desensitizing current (I_desens_) followed by a sustained current (I_sust_), consistent with the covalent modification of the channel. By contrast, MTSES was unable to activate G430C and to generate a sustained current (Fig 5C). Fig 5D shows the pH-dependency of the G430C activation by MTS-reagents for MTSET and MTSPTrEA. Both MTS-reagents generate a detectable sustained current at pH 7.8 that increases at pH values below 7.5, with a pH_0.5_ of 7.38 ± 0.02 and 7.43 ± 0.04 respectively. MTSET and MTSPTrEA differ in their efficiency to activate I_sust_ as shown by the current amplitude at pH 7.0 that represented, respectively, 0.41 ± 0.02 and 0.73 ± 0.03 fractions of I_max_ (Table 2).

**Table 2:** Relative efficiencies of MTS reagents to activate I_sust_ of ASIC1a-G430C at pH 7.4 and 7.0.

	Fractional I_sust_	
MTS-reagents (100μM)	pH 7.4	pH 7.0
MTSET	0.337 ± 0.058 n = 12	0.413 ± 0.033 n = 53
MTSPTrEA	0.394 ± 0.037 n = 13	0.725 ± 0.075* n = 10
MTSBT	0.177 ± 0.056 n = 5	0.726 ± 0.099* n = 15
MTSPT	0.704 ± 0.076 n = 5	0.845 ± 0.083 n = 10
MTSEA-biotin	0.023 ± 0.002 n = 10	0.148 ± 0.010* n = 26
MTSEA	nd	0.072 ± 0.024 n = 4
MTSES	nd	0.009 ± 0.003 n = 8

I_sust_ was elicited by addition of MTS-reagents at pH 7.4 or 7.0. Fractional I_sust_ represents the fraction of the maximal current elicited at pH 5.5 corresponding to I_sust_, as described in the protocol of Fig 5. I_sust_ elicited by MTSEA or MTSES could not be detected at pH 7.4 (nd), and was not significantly different from zero for MTSES at pH 7.0.

* denotes p<0.05 for values at pH7.0 versus pH7.4

Table 2 compares the efficiencies of the different MTS reagents to activate ASIC1 at pH 7.0 when the channel in a desensitized state, or at pH 7.4 when the channel is in a resting state. The positively charged MTS reagents were the ligands that most efficiently activated the channel, whereas the partially positively charged MTSEA or the neutral MTSEA-biotin were less efficient, and the negatively charged MTSES was totally ineffective. Although differences are observed in the sensitivities to MTS reagents of the desensitized or resting channels, all the MTS reagents except MTSES and MTSEA could activate the channel in both conformations.

**Fig 6:**
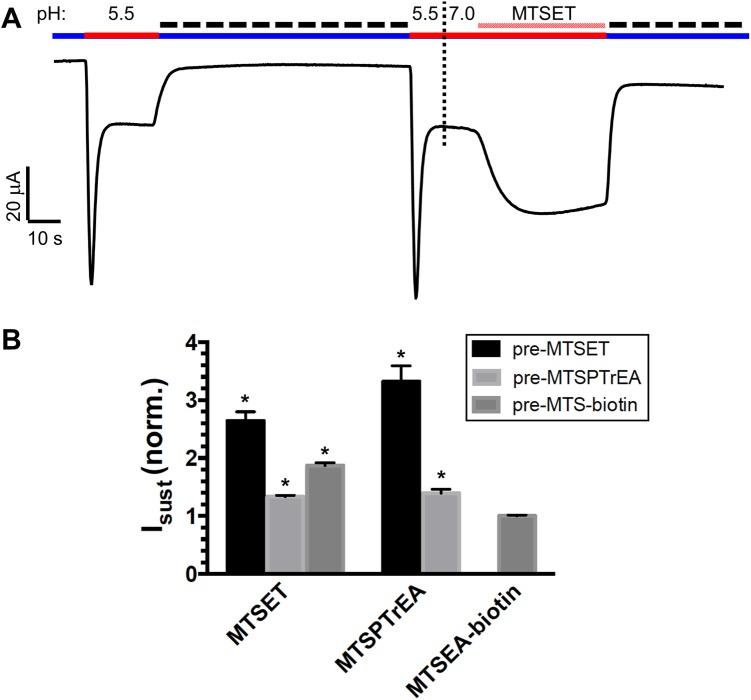
State-dependent stimulation of ASIC1a-G430C by MTS reagents. ASIC1a-G430C sustained current (I_sust_) was elicited by MTSET, MTSPTrEA, MTSEA-biotin in oocytes pre-incubated beforehand during 10 min. with 100 μM MTSET (n = 17), MTSPTrEA (n = 21), or MTSEA-biotin (n = 9). **A.** Representative current tracing of an oocyte expressing ASIC1a-G430C and pre-incubated with MTSET (100 μM). A first transient acidic pulse was performed from pH 7.8 (blue line) to pH 5.5 (red line); a second acidic pulse was repeated at pH 5.5, followed by the addition of MTSET (100 μM, crosshatching red line) at pH 7.0, before returning to baseline at pH7.8 in the presence of amiloride (300 μM, dashed line). **B.** I_sust_ elicited by MTSET (n = 22), MTSPTrEA (n = 19) or MTSEA-biotin (n = 6) (x-axis) during the second acidic pulse (see tracing in A.); I_sust_ elicited by the MTS-reagents were normalized for the I_sust_ values obtained before addition of the reagent. *denotes significance at p<0.01 using one-way ANOVA test using values obtained in oocytes pre-incubated and stimulated by MTSEA-biotin.

It is interesting to note that I_sust_ was larger when ASIC1-G430C was activated by MTSET (fractional I_sust_ = 0.337 ± 0.058, see Fig 5 and Table 2) than when the channel was beforehand modified by MTSET at pH 7.8 and then activated by H^+^ (fractional I_sust_ = 0.102 ± 0.005, see Fig 1 and Table 1). We hypothesized that ASIC1a-G430C in the open conformation was more sensitive to MTSET than in the closed resting state. We tested this hypothesis in the experiment described in Fig 6A. The oocytes expressing ASIC1a-G430C were pre-incubated with MTSET at pH 7.8, in the non-conducting state, and stimulated at pH 5.5 in the absence of MTSET to show both I_sust_ and I_desens_. A second and similar acidic pulse was repeated, followed by addition of MTSET at pH 7.0; the subsequent exposure of channels in the open conformation to MTSET resulted in a further increase in I_sust_. We interpret this effect as the result of the binding of additional MTSET molecules to G430C when the channel is in an open conducting state. We have tested MTSET, MTSPTrEA and MTSEA-biotin for their differential accessibility to G430C when the channel is in a non-conducting or conducting state (Fig 6B). The graph represents the fold increase in I_sust_ elicited by MTSET, MTSPTrEA or MTS-biotin after the second acidic pulse shown in Fig 6A. The data show that MTSET or MTSPTrEA could further increase I_sust_ of ASIC1a-G430C channels that had been modified beforehand (in a non-conducting state, pH 7.8) with MTSET, MTSPTrEA or MTSEA-biotin. However, for G430C bound to MTSEA-biotin in a non-conducting state, I_sust_ was not further stimulated by MTSEA-biotin when applied in the open conformation. These data suggest that the accessibility of G430C to MTSET or MTSPTrEA is more favorable in the conducting state.

**Fig 7:**
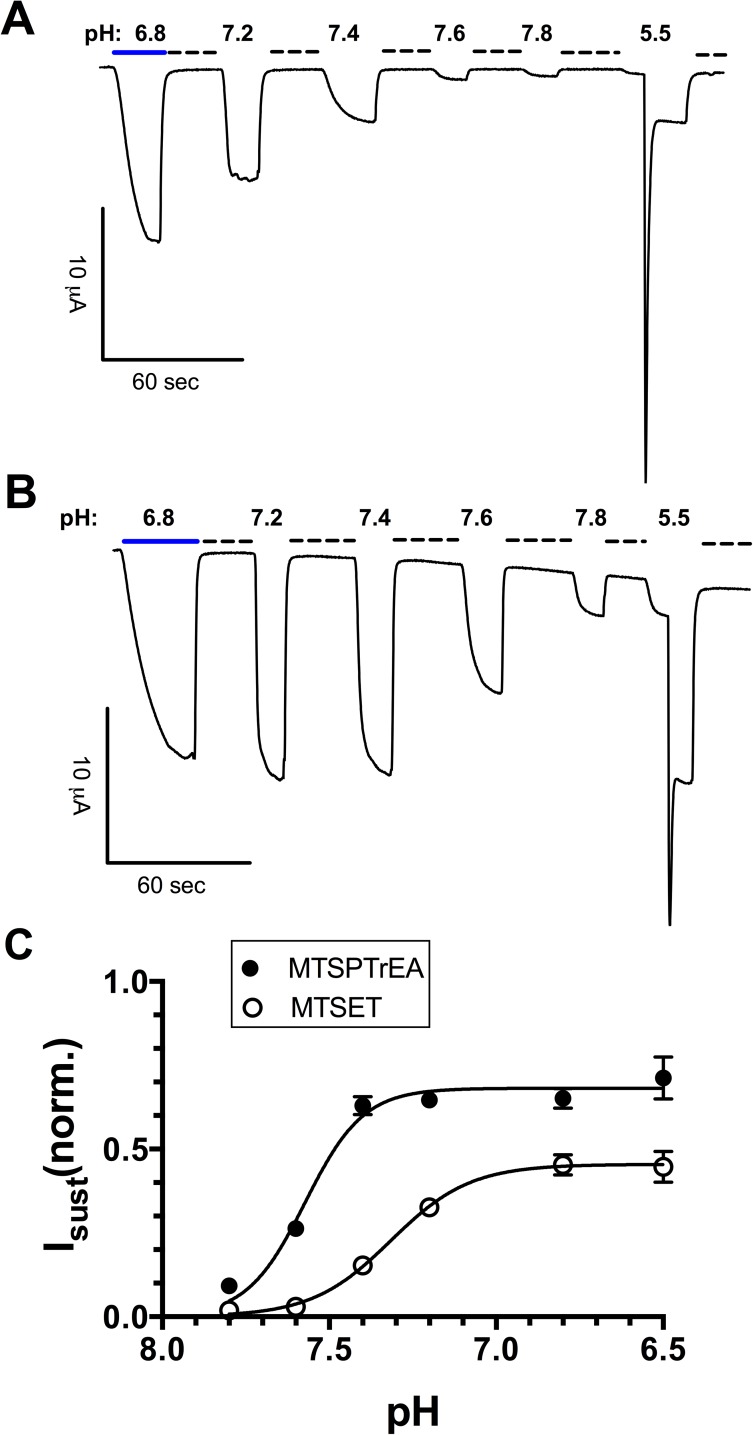
pH-dependence of the ASIC1a-G430C open state. **A-B**. ASIC1a currents were elicited by MTSET (A) or MTSPTrEA (B) at pH 6.8 (blue line), and measured at different pHs ranging from 7.2 to 7.8. Between each pH change ASIC1a current returned to baseline in the presence of amiloride at pH 7.8 (dotted line). At the end, ASIC1 currents were measured at pH 5.5 to assess Imax for current normalization **C**. pH-dependence between 6.5 and 7.8 of the ASIC1a-G430C I_sust_ after activation by MTSET (pH_0.5_ = 7.31, 95%CI:7.27–7.35, n = 33) or MTSPTrEA (pH_0.5_ = 7.57, 95%CI:7.54–7.60, n = 26). I_sust_(norm.) denotes I_sust_ normalized for maximal peak current elicited at pH 5.5. Symbols represent mean ± SE.

We have determined the pH sensitivity of the open state of ASIC1-G430C bound to MTSET or MTSPTrEA. The experimental protocol is illustrated in Fig 7A and 7B with representative tracings. The pH was set at 6.8 and ASIC1a was activated by MTSET or MTSPTrEA. ASIC1a current was subsequently measured at different pHs up to pH 7.8; between each pH change the current returned to baseline upon addition of amiloride at pH 7.8. Finally, a pH pulse to 5.5 was performed to assess Imax. The data in Fig 7C shows that the currents through ASIC1a in the open state bound to MTSET or MTSPTrEA are detectable up to pH 7.8, and the pH_0.5_ for sensitivity to pH was close to the physiological pH.

**Fig 8:**
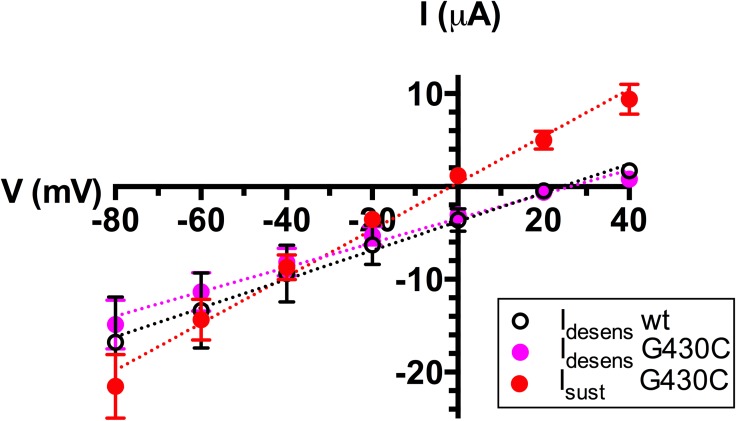
Current-voltage relations of ASIC1a wt and G430C. Peak desensitizing currents (I_desens_) triggered at pH 5.5 were measured for ASIC1a wt and ASIC1a-G430C at holding potentials ranging from -80 mV and + 40 mV. I_sust_ for ASIC1a-G430C was measured at pH 7.0 following 10 min pre-incubation at pH 7.8 with 100 μM MTSPTrEA. Reversal potentials, determined by linear regression analysis, were 23.42 ± 1.33 mV (n = 6), 26.15 ± 1.44 mV (n = 18) and -1.38 ± 0.92 mV (n = 12 p<0.001), respectively for the I_peak_ of ASIC1a-wt and ASIC1a-G430C, for the I_sust_ of the MTSPTrEA-modified ASIC1a-G430C.

So far, our experiments suggest that I_sust_ and I_desens_ are two distinct currents with different gating properties. We analyzed their respective conductive properties using current-voltage relations of I_desens_, of ASIC1a wt or ASIC1a-G430C, and of I_sust_ due to G430C activated by MTSPTrEA (Fig 8). The I_desens_ of G430C shows an I-V relation similar to that of wt ASIC1a, with positive reversal potentials indicative of a higher channel selectivity for Na^+^ over K^+^. By contrast, the I-V relation of I_sust_ for the MTSPTrEA-bound G430C has a reversal potential close to zero consistent with a non-selective current for Na^+^ and K^+^ ions. These results further suggest that two distinct channel open conformations are responsible for the I_desens_ and the I_sust_.

### Effects of MTS-reagents on ASIC2a

**Fig 9:**
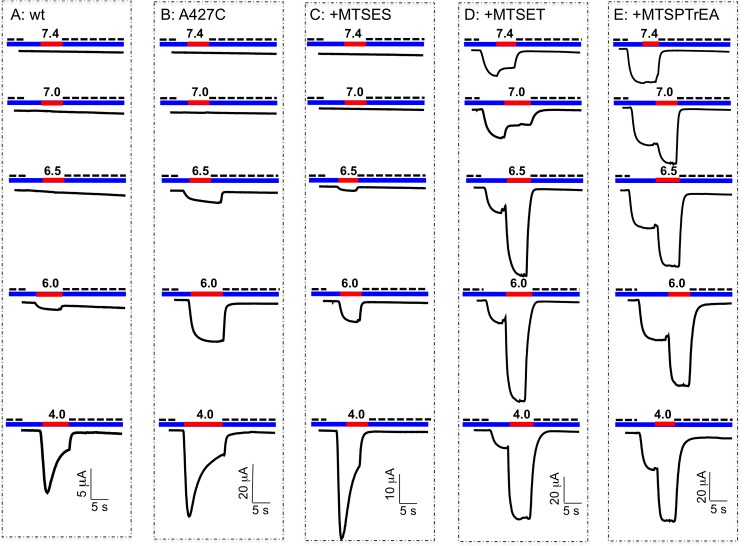
Representative recordings of ASIC2a-wt and ASIC2a-A427C. Oocytes expressing ASIC2a-wt or ASIC2a-A427C were perfused at pH7.8 (blue line); ASIC2a currents were elicited by a pH change to 7.4, 7.0, 6.5, 6.0 and 4.0 (red line) and currents subsequently blocked by perfusion with 300 μM amiloride (dashed line) at pH 7.8. Vertical alignment of the tracings from left to right show ASIC2a-wt (**A**), and ASIC2a-A427C (**B**) control currents, and ASIC2a-A427C currents after 10 min. pre-incubation with 100μM MTSES (**C**), MTSET (**D**), or MTSPTrEA (**E**).

We next addressed the question as to whether the sustained, non-desensitizing current, activated by MTS-reagents at physiological pH is specific for the ASIC1-G430C or whether it represents a common feature for ASIC channels under similar conditions. We reproduced the ASIC1a-G430C cysteine substitution in hASIC2a at the corresponding position (ASIC2a-A427C), and analyzed the transient and sustained currents activated either by protons or MTS-reagents. The representative traces in Fig 9 show that ASIC2a is activated at lower pH values than ASIC1a (Fig 9A); ASIC2a wt and ASIC2a-A427C open at pH 6.5 to 6.0 but, in contrast to ASIC1a, no clear desensitizing current was observed (Fig 9A and 9B). Under more acidic conditions however, the tracings show a current desensitization. Similar to ASIC1a-G430C, treatment with MTSES did not change ASIC2a-A427C currents (Fig 9C). Pre-incubation with either MTSET or MTSPTrEA did however elicit a sustained inward current at pH 7.8 upon removal of amiloride (Fig 9D and 9E). A dramatic increase in I_sust_ occurred below pH 7.0. Importantly, at all the tested pH values the current mediated by ASIC2a-A427C bound to MTSET or MTSPTrEA did not desensitize.

**Fig 10:**
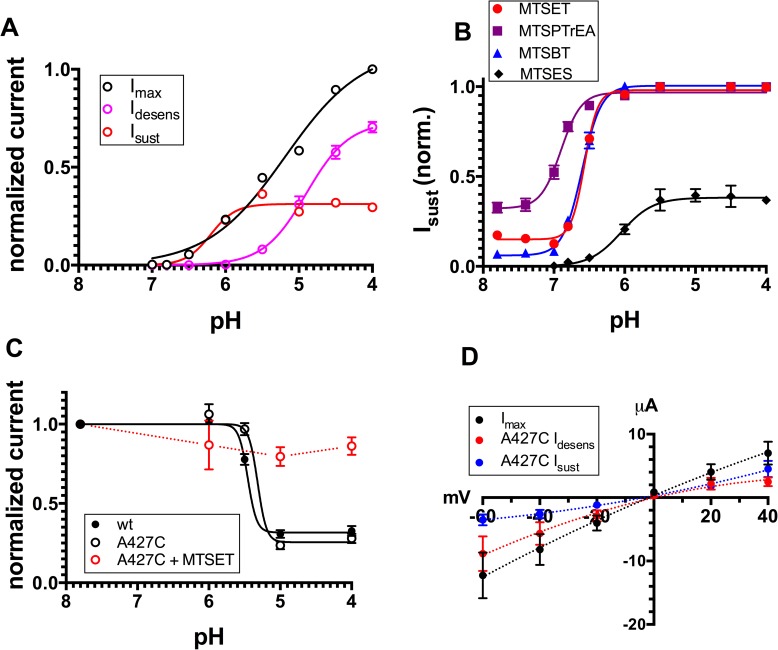
Sustained and desensitizing current of the ASIC2a-A427C mutant. **A.** pH-dependence of the maximal inward current (black), of the desensitizing current (purple) and the sustained current (red) of the ASIC2a-A427C. Each point represents the mean ± SE of 3 to 5 independent measurements. Curve fit of Imax represents the sum of the individual fits obtained for Idesens and Isust. **B.** pH-dependence of the I_sust_ recorded from oocytes expressing ASIC2a-A427C after pre-incubation with 100 μM of MTSET (red circles, MTSPTrEA (purple squares), MTSBT (blue triangles), or MTSES (black diamonds). Symbols represent means ± SE of 3 to 15 independent measurements. I_sust_ values were normalized for the maximal inward current elicited at pH 4.0; dashed lines represent best fit to current data. **C.** SSD determined for ASIC2a wt, A427C, and A427C incubated with MTSET; currents elicited by acidic pH 4.0, and measured after incubation at a conditioning pH ranging from 7.8 to 4.0. Symbols represent means ± SEM of 7 to 8 independent measurements. **D.** Current-voltage relations obtained for I_max_, I_sust_, and I_desens_ currents of ASIC2a-A427C unmodified by MTS-reagents; reversal potentials were (mean ± SE) 0.3 ± 1.8 mV, -8.7 ± 2.4 mV and 5.8 ± 1.3 mV (p<0.01), respectively (n = 5). Similar values were obtained for ASIC2wt for these three currents, respectively 1.2 ± 4.5 mV, -7.1 ± 4.0 mV and 12.5 ± 4.5 mV (n = 5).

Graphs in Fig 10 illustrate the main characteristics of the ASIC2a-A427C currents before and after modification by MTS-reagents. ASIC2a-A427C shows both I_sust_ and I_desens_. The I_sust_ of A427C is smaller in magnitude than I_desens_ at pH 4, and neither current was detected at pH 7.0 and above (Fig 10A). The pH_0.5_ for activation of I_sust_ was more alkaline than for I_desens_, but still remains below neutral pH. After modification by MTSET, MTSPTrEA, or MTSBT, I_sust_ could be detected above pH 7.0 and further increased up to values corresponding to the maximal current of ASIC2a-A427C when the pH drops below 7.0 (Fig 10B). MTSES was without effect on the I_sust_, but induced a slight alkaline shift of I_desens_ (Table 3).

**Table 3:** pH dependency of current activation and steady state desensitization (SSD) of ASIC2a-A427C bound to MTS-reagents.

	I_max_ (μA)	I_sust_ pH_0.5_	Fractional I_sust_	I_desens_ pH_0.5_	SSD pH_0.5_
ASIC2a (n = 12)	8.23± 1.75	5.68 (5.53—5.83)	0.404 ± 0.019	4.96 (4.88-5.04)	5.45 (5.43-5.48)
ASIC2a-A427C (n = 15)	27.39± 3.95	6.20* (6.05-6.36)	0.312 ±0.016	4.90 (4.81-4.99)	5.32 (5.16-5.47)
A427C+MTSES (n = 6)	46.20± 7.63*	6.05* (5.96-6.14)	0.384±0.014	5.24* (5.17-5.31)	nd
A427C+MTSET (n = 15)	74.23± 3.28*	6.57* (6.55-6.59)	1.0	nd	nd
A427C+MTSBT (n = 15)	75.90± 4.49*	6.60* (6.58-6.63)	1.0	nd	nd
A427C+MTSPTrEA(n = 14)	66.07± 5.85*	6.90* (6.86-6.94)	1.0	nd	nd

Currents were elicited at pH 4.0. The values correspond to data shown in Fig 8 and represent mean ± SE; values in parenthesis are 95% confidence interval values obtained for the fit. Legends have the same meaning as in Table 1. ‘nd’ denotes ‘non detectable’.

* denotes statistical significance (p<0.05) compared to ASIC2a wt or to ASIC2a-A427C (one-way ANOVA).

The pH dependences of the SSD for ASIC2a and the ASIC2a-A427C mutant were quite similar with a pH_0.5_ around 5.4, and no SSD could be observed after modification by MTSET at a pH as acidic as 4.0 (Fig 10C). The IV relations for I_max_, I_desens,_ and I_sust_ currents measured for the ASIC2a-A427C in the absence of MTS-reagents, show a small but significant difference (p<0.01) between the reversal potentials of I_sust_ (-8.8 ± 2.4, n = 5) and I_desens_ (5.8 ± 1.3, n = 5). This suggests that, as for ASIC1a-G430C, there is a slight difference in the ionic selectivity of these two conducting states (Fig 10D).

**Fig 11:**
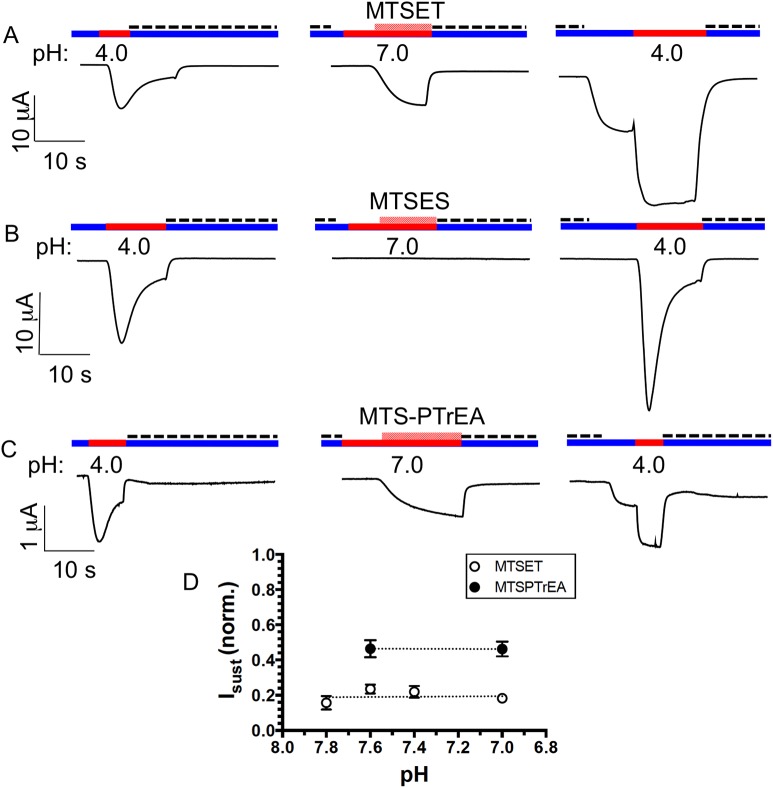
Activation of ASIC2a-A427C by MTS-reagents at neutral pH. Oocytes expressing ASIC2a-A427C were subjected to a protocol similar as in Fig 5A. After an initial current pulse elicited at pH 4.0, ASIC2a-A427C was activated at pH 7.0 by 100 μM of MTSET (**A**), MTSES (**B**), or MTSPTrEA (**C**). The recording was terminated by a final pH pulse at pH 4.0. Blue line corresponds to pH 7.8, the red line to acidic pH 7.0 or 4.0, dashed line to the addition of 300 μM amiloride. **D.** pH-dependence of I_sust_ normalized for the maximal current elicited at pH 4.0 elicited by MTSET (n = 4–13) or MTSPTrEA (n = 6–10).

Finally, the representative tracings on Fig 11 show that, as for ASIC1a-G430C, ASIC2a-A427C can be activated at physiological pH by MTSET (A) or MTSPTrEA (C), but not by MTSES (B). After a first pulse to pH 4.0, the pH was set at 7.0, and the addition of MTSET triggered an amiloride-sensitive current with slow activation kinetics. After returning to pH 7.8, removal of the amiloride shows the persistence of an ASIC2a current that can be further stimulated at pH 4.0. MTSES was without effect on channel activation or desensitization except for an increase in the magnitude of the peak current elicited at pH 4.0 (Fig 11B, right panel). Furthermore, the I_sust_ elicited by MSTET or MTSPTrEA was insensitive to pH between 7.8 and 7.0, the magnitude of I_sust_ being larger for MTSPTrEA than for MTSET (Fig 11D). In this pH range, the I_sust_ elicited by MTSET and MTSPTrEA closely matches the current elicited by protons on ASIC2a-A427C that has been previously modified by the same MTS-reagents (Fig 10).

In summary, our results show that ASIC1a and ASIC2a exhibit 2 distinct types of currents, a sustained, non-desensitizing current (I_sust_) and a desensitizing current (I_desens_), depending on the activating ligand. The I_sust_ currents triggered by cationic MTS-reagents differ from I_desens_ by its pH-dependence, ionic selectivity, and relative magnitude. The I_sust_ triggered by the MTS-reagent is ligand specific.

## Discussion

In this work we have analyzed the pH dependency of activation and desensitization of ASIC1a and ASIC2a bound covalently to a ligand in the extracellular vestibule. The goal was to better understand the ASIC1a channel behavior in a bound state with a non-proton ligand under physiological, non-acidic pH conditions. The ASIC1a-G430Cand ASIC2a-A427C mutants behave similarly to their wild type counterparts, but can be covalently modified by MTS-reagents of different size and carrying different charges. This experimental approach artificially reproduces a site-specific binding of extracellular ligands in the vestibule of the channel. The covalent nature of the binding of MTS-reagents to the cysteine introduced in ASIC1a and ASIC2a differs however from the reversible interactions with specific pharmacological or natural ligands. Such covalent binding of the MTS-reagent has the advantage to allow an unambiguous study of the bound state of the channel.

**Fig 12:**
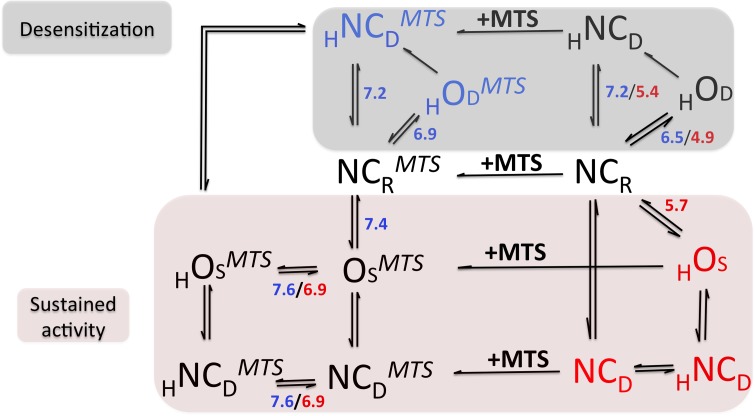
Kinetics model for ASIC1a and ASIC2a activity underlying sustained and desensitizing currents. The different channel conformation states represented in this model have been identified in our experiments. In black are the conformation states common to ASIC1a and ASIC2a, in blue conformation states observed only for ASIC1a, in red conformation states observed only for ASIC2a. Numbers denote the pK_a_ values taken for our experiments of the equilibrium reaction between 2 states, in blue for ASIC1a, in red for ASIC2a. Non-conducting ASICs at pH 7.4 in a resting state (NC_R_) can undergo either desensitization (I_desens_, grey area) or a sustained activity (I_sust_, pink area), depending on the external pH and/or binding of MTSPTrEA (MTS). The NC_R_ is in equilibrium with the desensitized state _H_NC_D_ and with the open state _H_OD, the latter being in a non-equilibrium with the _H_NC_D_ state. The pK_a_ values for these reactions (blue for ASIC1a, red for ASIC2a) were determined in our experiments. In ASIC2a, NC_R_ state is in equilibrium with a desensitized state NC_D_ and an open state _H_O_S_ depending on the pH; by contrast to ASIC1a, this _H_O_S_ is in equilibrium with a desensitized _H_NC_D_. Binding of MTSPTrEA to resting, desensitizing or open states (NC_R_^MTS^, _H_NC_D_^MTS^, _H_O_D_^MTS^) promotes a channel open state in equilibrium with the desensitized states to trigger I_sust_.

We found that ASIC1a, like ASIC2a, exhibit two distinct types of currents depending on whether the channels are in a bound or unbound state with MTS-reagents. These two distinct currents differ according to their pH_0.5_ for activation, their apparent kinetics of activation, their desensitization and their ion conductive properties. Based on our results on the pH dependency of the I_sust_ and I_desens_ recorded from ASIC1a-G430C and ASIC2-A427C channels, free or bound to our prototypical MTS-reagent MTSPTrEA, we propose in Fig 12 a simple model for channel transitions between conducting (O states = open states) and non-conducting states (NC states) to recapitulate our experiments. We have constructed this model on the well-established concept that ASIC channels, when activated by protons, transit between two non-conducting states, namely a resting and a desensitized state, through a transient open state [1]

### Channel desensitization

Based on the currents elicited by ASIC1a, it is generally accepted that, depending on the extracellular pH, this channel transits between 3 conformational states, namely a non-conducting resting state, an open state, and a desensitized state [1]. The current recording for ASIC1a-G430C shown on Fig 2A is consistent with this established model. As illustrated in Fig 12, at pHs ranging from 7.4 to 7.0, ASIC1a is in equilibrium between a non-conducting, resting state (NC_R_) and a non-conducting desensitized state (_H_NC_D_). The transition between these two non-conducting states called steady-state desensitization (SSD), shows a steep pH-dependency with a pKa around 7.2 that is similar for ASIC1 wt and ASIC1a-G430C (see in Fig 4 and Table 1). Only when the external pH drops below pH 7.0, this transition from the resting (NC_R_) to the desensitized state (_H_NC_D_) occurs via a transient open state (_H_O_D_). This transition from a resting to an open state is called channel activation and shows a similar pH-dependency (pH_0.5_) around 6.4 for ASIC1 wt and ASIC1a-G430C (S2 Fig and Table 1). The transition between the open _H_O_D_ and desensitized _H_NC_D_ states is irreversible as shown by the rapidly desensitizing ASIC1 current. We have no indication that this irreversible _H_O_D_ to _H_NC_D_ transition is pH-dependent.

The pH-dependent desensitizing current I_desens_ of ASIC2a and ASIC2a-A427C (Fig 9A) reflects similar transitions between resting (NC_R_), open (_H_O_D_), and desensitized (_H_NC_D_) states. As for G430C mutation in ASIC1a, the corresponding mutation A427C does not affect these transitions in ASIC2a (Fig 9B). However, 3 important differences exist between these two channels. First, the pH_0.5_ for channel activation is lower for ASIC2a than for ASIC1a (see S2 Fig and Fig 10A). Second, at pHs ≥6 ASIC2a elicits a sustained current I_sust_; only at pH<6, a desensitizing current is observed along with the I_sust_ (Fig 9A and 9B). We interpret these results as the presence of at least two transitions from a resting channel NC_R_ towards an open state. One open state (_H_O_S_) is in equilibrium with a desensitized state (_H_NC_D_) responsible for I_sust_. A second, protonated open state (_H_O_D_) undergoes the irreversible desensitization which is responsible for I_desens_. The pH-dependence of these two transitions is determined by the respective pH-dependences of activation of I_desens_ and I_sust_ (Fig 10A and Tables 1 and 3). It should be noted that the ASIC2a-A427C mutation results in a significant alkaline shift of the pH-dependence of I_sust_ activation from pH_0.5_ = 5.7 to 6.2; this represents the only functional difference between the wt and the mutant ASIC2a. Third, the pH-dependency of the steady state desensitization of ASIC2a is shifted to more acidic pHs compared to ASIC1a (see Tables 1 and 3).

After ASIC1a incubation with MTS reagents, the pH-dependency of the desensitizing current is slightly shifted towards a more alkaline pH (see Fig 3 and Table 1). This shift in the pH_0.5_ for activation of I_desens_ was observed with all MTS-reagents bound to ASIC1a, including MTSES (Fig 3). This is illustrated in Fig 12 by the transition of the ASIC1a-G430C bound to MTSPTrEA from its non-conducting resting state (NC_R_^MTS^) to the desensitized state (_H_NC_D_^MTS^) via the open state (_H_O_D_^MTS^) with a pH-dependence of pH_0.5_ = 6.9 (Table 1). The channel open state (_H_O_D_^MTS^), bound to MTS, undergoing desensitization seems to be specific to ASIC1a, since ASIC2a bound to MTS reagents does show any desensitizing current (Figs 9D, 9E and 10C). Interestingly, the pH_0.5_ for the steady state desensitization (SSD) of ASIC1a is not affected by the binding of MTS (pH_0.5_ = 7.2). Thus, the pH-dependence of ASIC1a activation of I_desens_ can be modulated by ligands without concomitant changes in the pH_0.5_ of SSD. These observations suggest that ASIC1a activation and desensitization by protons represent two independent processes.

### Channel sustained activity

We have already discussed I_sust_ of ASIC2a elicited by protons, and we interpreted this current as resulting from a new equilibrium between the channel open state _H_O_S_ and a non-conducting desensitized state _H_NC_D_ (Fig 12). This equilibrium was not found for ASIC1a in an MTS unbound state. Binding of MTSPTrEA to resting ASIC1a at pH 7.4 triggers a I_sust_ at pH values as high as 7.8, the maximal activation being obtained at around pH 7.2 (pH_0.5_ = 7.43, see Fig 5D). This is illustrated in Fig 12 by the covalent binding of MTS to resting ASIC1a NC_R_ and the new equilibrium between NC_R_^MTS^ and the open state O_S_^MTS^ with a pKa of 7.4 (Fig 5D). Since MTSPTrEA can trigger I_sust_ below pH 7.2 (Fig 5D), it also binds the desensitized state _H_NC_D,_ allowing _H_NC_D_^MTS^ to be in equilibrium with the open _H_O_S_^MTS^. This transition shows no pH-dependency as shown by the I_sust_ saturation at pHs below 7.2 (Fig 5D). We have tested the pH-dependence of the ASIC1a open state bound to MTS and we could show that I_sust_ activated by MTSPTrEA or MTSET (Fig 7A and 7B) was stimulated by acidic pH in a physiological range (pH_0.5_ = 7.6, Fig 7C). This is illustrated in Fig 12 by the pH-dependence of the equilibrium between O_S_^MTS^ to _H_O_S_^MTS^.

MTS binds ASIC2a-A427C in the essentially resting, non-conducting state (NC_R_) when incubated at pH > 7.0 at which no ASIC2a current is detected (Fig 9B). However, at pH < 7.0 at which an I_sust_ is detected, MTS likely binds to both the resting (NC_R_) and the desensitized (NC_D_) states, which are in equilibrium with the open state (O_S_), to further increase I_sust_. For ASIC2a-A427C, we observed no pH-dependency of the I_sust_ for pH > 7.0. However, when the pH drops below 7.0 we observe a sharp increase in I_sust_ (Fig 10B). We have translated this observation into our model (Fig 12) as the absence of pH-dependency of the equilibrium between the NC_R_^MTS^ or NC_D_^MTS^ states and O_S_^MTS^; protonation of the open ASIC2a-A427C O_S_^MTS^ state into _H_O_S_^MTS^ further increases I_sust_ (pKa = 6.9, Fig 9B).

In conclusion, our model in Fig 12 recapitulates our experiments and stresses the fact that ASIC1a and ASIC2a are able to function in both a desensitizing and a non-desensitizing modes. The desensitizing mode is triggered by protons specifically, whereas binding of non-proton ligands allows the channels to escape desensitization and to function in a non- desensitizing mode. In the latter mode of activity, the channels retain a pH-dependency within pH ranges close to the physiological pH. In our model, the pH-dependencies of the equilibria between the different conformational states bound to MTS correspond to data from assays with MTSPTrEA. Our experiments revealed a ligand-specificity among the MTS-reagents for their effects on the pH-dependence of I_sust_ and I_desens_, as well as for their relative magnitudes. Our model holds for all MTS-reagents except for MTSES, which does not trigger a non-desensitizing current, and for the wt ASICs and their Cys mutants.

### Evidence for two gating sites in ASIC1a

In our model we postulate the existence of at least two discrete open states for ASIC1a and ASIC2a, one in non-equilibrium and one in equilibrium with a desensitized state, that account, respectively for the desensitizing and the sustained currents. This opens the possibility of the participation of 2 independent gating sites on ASIC1 and ASIC2.

Protons stabilize the ASIC1 desensitized state directly or via a transient open state. These two independent pathways towards channel desensitization show different pH sensitivities, possibly reflecting protonation of specific amino acid residues according to their local pK_a_ values. Several mutations of acidic residues introduced into different sub-domains of the extracellular loop of ASIC channels, such as the acidic pocket, the palm, and the wrist, have been shown to shift the pH-dependence desensitizing current activation and/or the SSD [16–20]. There is no single site identified as responsible for the protonation-induced channel desensitization. It is possible that an extended proton screening of negative charges at the surface of the extracellular domain is responsible for ASIC desensitization.

From our experiments with cationic MTS-reagents, the N-terminal start of the second transmembrane segment of ASIC1 and ASIC2 constitutes a potential binding site in the pore vestibule for the generation of sustained, currents. Channel opening occurs at physiological pH, originating from either a resting or a desensitized state. A specificity for MTS-ligand to trigger sustained ASIC1a activation was previously recognized [11]. The size of the ligand may play a role in sustained activation of ASIC1, as suggested by MTSPTrEA being bulkier and more efficient that MTSET to induce a sustained current; the same seems to apply for MTSEA-biotin compared to MTSEA. The number of MTS molecules bound per ASIC channel likely determines the amplitude of this sustained current, further supporting the functional relevance of the volume occupied by the ligand in the channel vestibule. We have observed that the open ASIC1a is more sensitive to MTSET for inducing I_sust_ than the close resting state (Fig 6). To a lesser extent, this was also the case for MTSPTrEA but not for MTSEA-biotin. This suggests that more than one molecule of MTSET is needed to bind into the pore vestibule to induce a maximal amiloride-sensitive I_sust_. This implies as well that the pore vestibule is large enough to accommodate several MTSET molecules without impairing the access of amiloride to its blocking site. Finally, the charge carried by the MTS-reagents in another important factor: the negatively charged MTSES is inefficient in inducing I_sust_ and MTSEA, which is only partially positively charged at pH7.4, is less efficient than the purely cationic MTSET.

### Non-proton activation of ASIC

Reversible binding of GMQ, a synthetic guanidinium derivative, activates ASIC3. GMQ activates a pH-dependent sustained ASIC3 current with a shift towards alkaline pHs; at a pH below 7.0, channel desensitization can still be observed [9]. The GMQ effect is specific for ASIC3, since it does not activate a sustained current in ASIC1a [21].

Previous studies have identified the initial N-terminal part of the TM2 as a binding site for ligands that are able to activate and open the channel at neutral pH. They showed that Zn^2+^, MTS reagents, and even amiloride are able to activate or generate constitutive currents by ASIC2 mutants with cysteine or valine substitutions of the glycine corresponding to G433 in ASIC1a [22, 23]. Tolino et al. previously observed that positively charged MTS reagents trigger G430C channel opening [11]. Based on a double mutant analysis of the effect of MTS, the authors provided a mechanistic interpretation for channel opening involving a rotation of the second transmembrane helix. This study did not discuss the fact that the channel does not desensitize in this open conformation. Covalent binding of an azobenzene to cysteines engineered at positions G430 or I428 in the vestibule triggers an ASIC1 opening that is persistent upon light-induced isomerization of the ligand. The interpretation was that, upon isomerization, the azobenzene group pushes apart the transmembrane helices leading to channel opening.

These studies, together with our work, identify the external vestibule of the channel pore as a potential binding domain for non-proton ligands to activate in a sustained manner ASIC channels at physiological pH.

### Physiological implications

In the absence of clear evidence for specific endogenous ligands that activate ASIC, most of the functional studies have considered protons as the physiological activators of these channels. At a physiological pH ranging from 7.4 down to 7.0, protons essentially desensitize the channel and only at more acidic pH values, channel desensitization transits via a shortly-lived channel opening.

A recent work has proposed that protons fulfill the criteria of a neurotransmitter, since they are released upon presynaptic stimulation, leading to a transient acidification of the synapse [7]. Changes in the buffering capacity within the synaptic cleft modulate ASIC-dependent post-synaptic excitatory currents [8]. However, there is no direct evidence that the transient acidification upon release of synaptic vesicles is sufficient for ASIC1a activation. Such acidification was indirectly quantified to represent a drop of only 0.2 pH unit [24].

It should be pointed out that fast channel desensitization is a rather uncommon behavior among the members of the ENaC/degenerin ion channel family. In fact ASICs are the only channels that rapidly desensitize, unlike other subfamily members such as ENaC, FaNaCh, and the drosophila homolog Ripped Pocket (RPK) channels that show either a constitutive or a ligand-gated sustained activity [1, 14, 25]. Many channels of this family are inactive when expressed in heterologous cell systems, possibly because their specific ligands have not yet been identified.

In accordance with previous studies, our work shows that ASIC1 and ASIC2 can be activated by specific non-proton ligands binding in the pore vestibule of the channel, at neutral pH. The resulting sustained ASIC activity can be modulated by protons under conditions close to physiological pH, as in the case of ASIC1a. The relevance *in vivo* of this sustained ASIC activity remains presently hypothetical until physiological endogenous ligand for sustained channel activation at neutral pHs will be identified. The demonstration that ASIC1 and ASIC2 can function at neutral pH without desensitization, provides however a proof of principle that protons should not be considered as the only possible endogenous activator of ASIC channels. Further research is needed to identify non-protons ligands in order to better understand the physiological role of ASIC channels.

## Materials and methods

### Electrophysiology

*Xenopus leavis* frogs were anesthesized using MS222 0.2%. A 0.8 cm incision was performed in the abdominal wall to removed the oocytes. Frogs were used several times for collection of oocytes. The procedure was approved by the ethical committee of the veterinary office of the Kanton. Healthy stage V and VI *Xenopus laevis* oocytes were pressure-injected with 10 ng of cRNA coding for human ASIC1a and ASIC2a. Oocytes were then maintained in a standard MBS medium before experiments. Electrophysiological measurements were performed 24h after oocyte injection with hASIC1a or hASIC2a cRNAs [26]. When indicated, oocytes were incubated for 10 min. with MTS-reagents (100 μM) at pH 7.8 before current measurements. ASIC currents were measured using the two-electrode voltage-clamp (TEV) as described previously [26]. Macroscopic ASIC1a and ASIC2a currents were elicited by rapid changes in extracellular pH from 7.8 to a pH defined by the experimental protocol, after removal of amiloride from the bath (300 μM). For each experiment, the initial acidic pH pulse lasted 10 s., and a 40 s. time-period for channel recovery was introduced before the next stimulatory phase, triggered either by MTS reagents or protons. ASIC currents elicited by MTS-reagents (100 μM) were performed by adding the reagent directly into the bathing solution at the pH defined by the protocol and after removal of amiloride (300 μM) from the bath. Steady state desensitization (SSD) was determined by perfusing the oocyte in a conditioning solution for 40 s. at the desired pH ranging between pH 8 and 4, before measuring the ASIC current elicited at pH 5.5. Accessibility and covalent modification of G430C by MTS-reagents was assessed in competition experiments in which MTS reagents were used to antagonize the block of ASIC-G430C by BMOE, a maleimide crosslinker that covalently conjugates sulfhydryl groups of cysteines at position G430 [15]. Oocytes were incubated in MBS at pH 7.8 in the presence of 10 μM of either MTSET, MTRPTrEA, MTSEA-biotin, or MTSES with increasing time of exposure ranging from 0 to 6 min. Exposure to MTS-reagent was terminated by washout of the reagents in the presence of 100 μM of cysteine. Then the oocytes were incubated during 5 min. in the presence of 2 mM BMOE and the ASIC1a amiloride-sensitive current elicited at pH 5.5 was measured at -80 mV. Current values were normalized for ASIC1a current measured in control oocytes being previously exposed during 10 minutes to 10 μM of MTSET, MTSPTrEA, MTSEA-biotin or MTSES, but not to the blocker BMOE.

### Definitions

Maximal current (I_max_) is defined as the maximal peak inward current sensitive to amiloride and elicited by protons at supra-maximal concentrations, i.e. pH 5.5 for ASIC1a and pH4.0 for ASIC2a. The sustained current (I_sust_) is defined as the residual amiloride-sensitive current measured >5 seconds after the maximal inward peak current. The desensitized current (I_desens_) was calculated by subtracting the sustained current from the total current.

### Molecular biology

The hASIC1a G430C mutation has been previously described [15]. pSD(BS)-H8-hASIC2a was generated by subcloning a hASIC2a amplicon into the SalI-SpeI linearized pSD(BS)-H8, a cloning vector with a sequence encoding an octahistidine tag inserted between the XhoI and SalI sites of pSD(BS). The ASIC2a A427C mutation was obtained by replacing the MunI-BclI insert from pSD(BS)-H8-hASIC2a by a synthetic DNA (Eurofins genomics, Ebersberg bei München, Germany) containing the corresponding codon replacement (GCC -> TGC). All vectors were verified by sequencing.

### Solutions and products

MBS solution contains (mM): NaCl 85, HEPES 10, NaOH 4.08, NaHCO_3_ 2.4, KCl 1, MgSO_4_ 0.82, CaCl_2_ 0.41, Ca(NO_3_) 0.33). Current measurements were done in a solution containing (mM): NaCl 120, HEPES or MES 10, MgCl_2_ 2, and the final pH adjusted with N-methyl-D-glucamine. Amiloride was purchased from Sigma. MTSEA (2-Aminoethyl Methanethiosulfonate hydrobromide), MTSET ([2-(Trimethylammonium)ethyl]methanethiosulfonate bromide), MTSES (Sodium (2-Sulfonatoethyl)methanethiosulfonate), MTSPTrEA (3-(Triethylammonium)propyl methanethiosulfonate bromide), MTSEA-Biotin (N-Biotinylaminoethyl methanethiosulfonate), MTSBT ([4-(Trimethylammonium)butyl] methanethiosulfonate bromide), MTSPT ([3-(Trimethylammonium)propyl] methanethiosulfonate bromide) were purchased from Toronto Research Medical, Canada.

### Statistical analysis

Current values versus pH obtained experimentally were fitted using a single component non-linear regression analysis with variables including the pH of ½ maximal current activation (pH_0.5_), Hill coefficient, minimal and maximal current levels. Statistical significance was defined as p<0.05 using either standard student’s t-test, or ANOVA when appropriate

## Supporting information

S1 Fig Structures of the MTS-reagents tested in this study. (TIF)

S2 Fig pH-dependency of the current peak of the ASIC1-wt, ASIC1a-G430C, and ASIC1a-wt after pre-incubation with MTSET. Currents were normalized for the maximal peak current measured at pH 5.5. Symbols represent mean ± SEM of 10 to 49 independent measurements.
(TIF)
