# Supplementary figures and images for: Proton and non-proton activation of ASIC channels
Source: PLoS One. 2017 Apr 6;12(4):e0175293. doi: 10.1371/journal.pone.0175293 (PMC5383329; doi:10.1371/journal.pone.0175293)

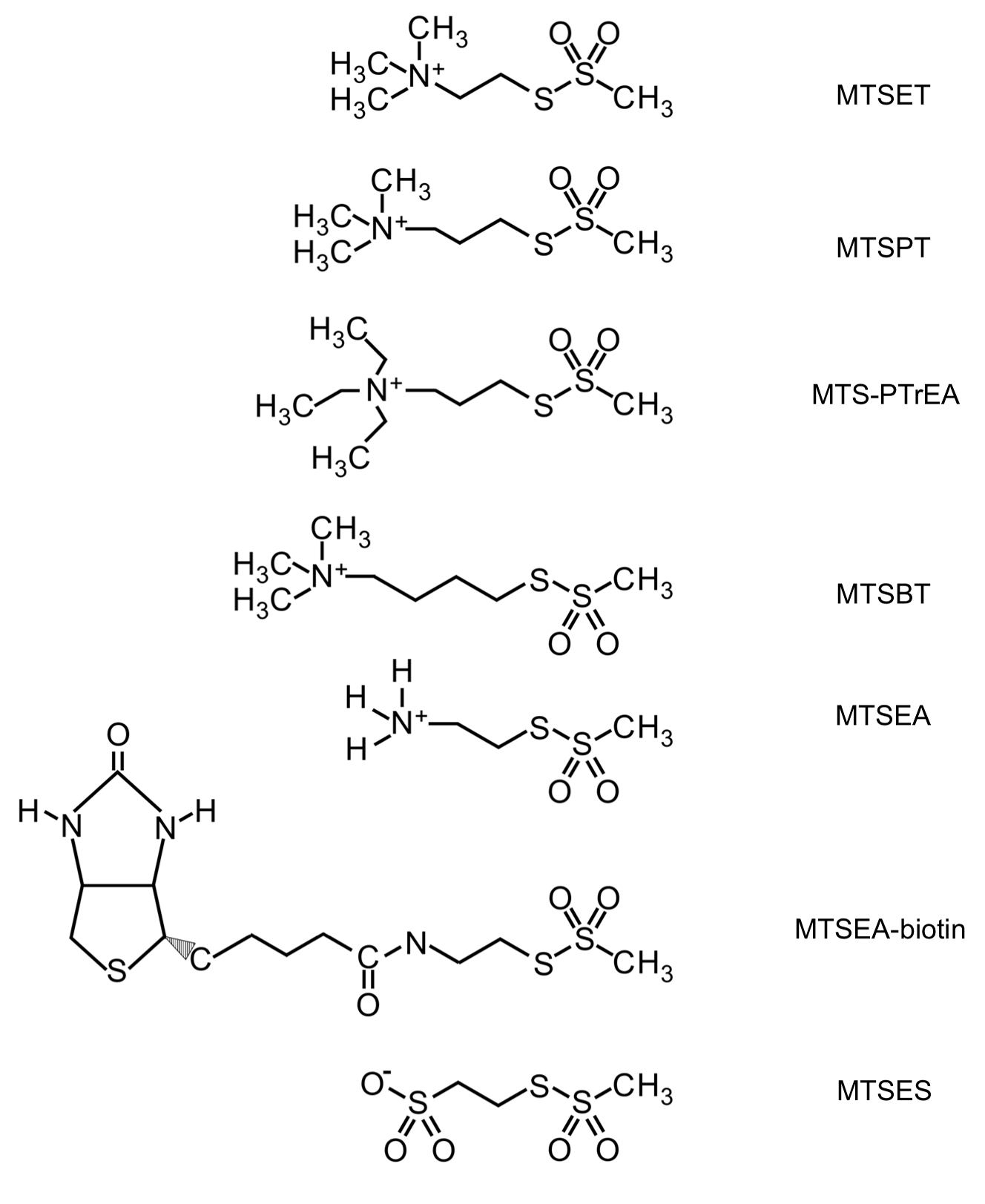

Supplement: S1 Fig — (TIF) [file pone.0175293.s001.tif]

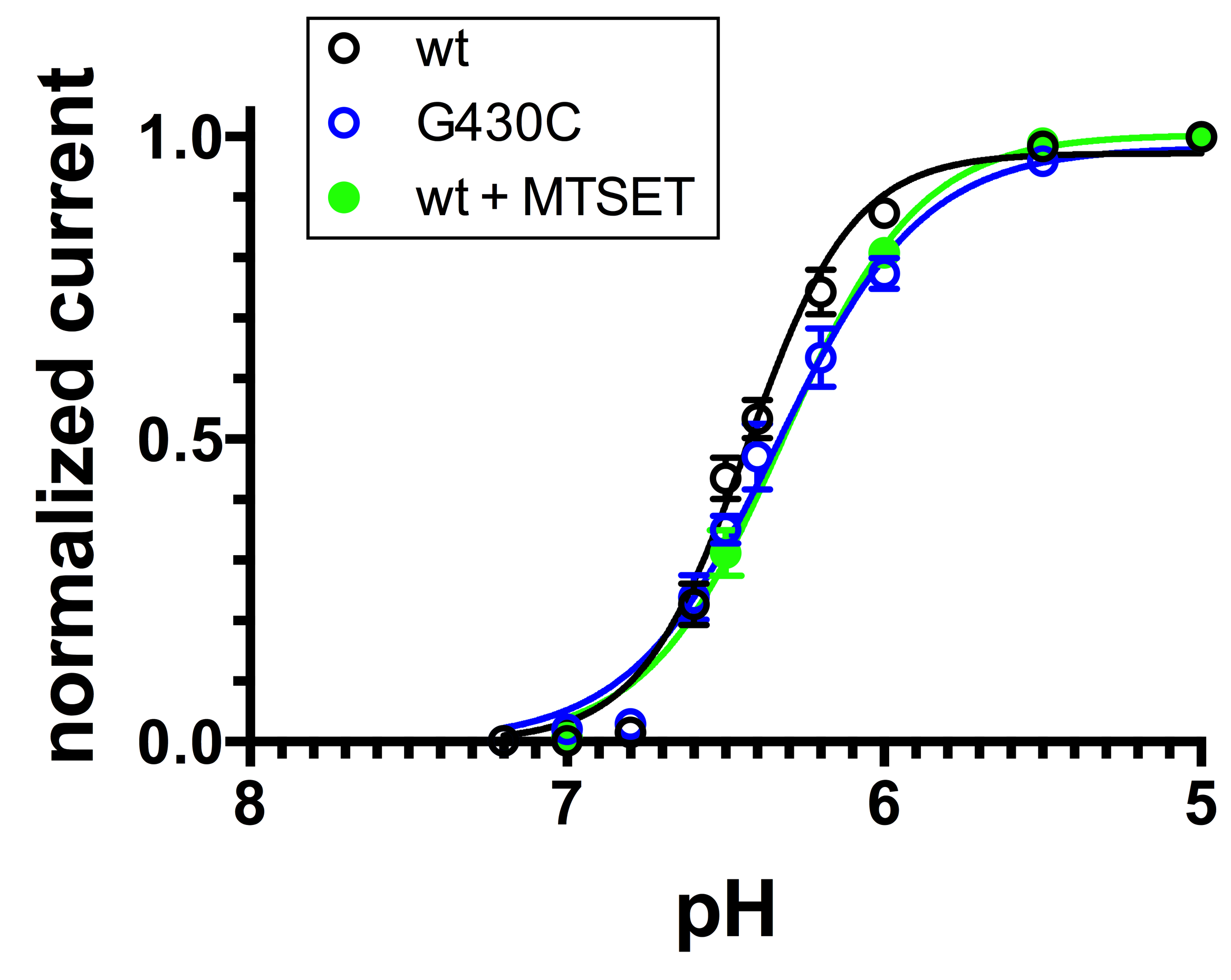

Supplement: S2 Fig — Currents were normalized for the maximal peak current measured at pH 5.5. Symbols represent mean ± SEM of 10 to 49 independent measurements. (TIF) [file pone.0175293.s002.tif]
